# Epidermolysis bullosa and global challenges in translational research for rare diseases

**DOI:** 10.31744/einstein_journal/2026Suppl_2ED0001

**Published:** 2026-03-20

**Authors:** Priscila Keiko Matsumoto Martin, Laurent Ketlen Leão Viana, Mel Dominici Sanfins

**Affiliations:** 1 Hospital Israelita Albert Einstein São Paulo SP Brazil Hospital Israelita Albert Einstein, São Paulo, SP, Brazil.; 2 Debra Brasil Blumenau SC Brazil Debra Brasil- Blumenau, SC, Brazil.; 3 Hospital Israelita Albert Einstein Faculdade Israelita de Ciências da Saúde Albert Einstein São Paulo SP Brazil Faculdade Israelita de Ciências da Saúde Albert Einstein, Hospital Israelita Albert Einstein, São Paulo, SP, Brazil.

Rare diseases collectively affect hundreds of millions of individuals worldwide; however, each condition individually faces substantial barriers to research, innovation, and equitable access to therapies. Accelerating and promoting research in rare diseases is therefore not only a scientific necessity but also a public health and ethical imperative. Limited funding, small patient populations, fragmented expertise, and complex regulatory and reimbursement pathways frequently delay the translation of scientific advances into tangible clinical benefits, particularly outside high-income regions. Even when innovative therapies receive regulatory approval, access often remains restricted to a small number of countries, reinforcing global disparities. Epidermolysis Bullosa (EB) exemplifies these challenges while simultaneously serving as a paradigmatic model for advances in gene therapy, regenerative medicine, and precision therapeutics.

Epidermolysis Bullosa is a group of rare, inherited genetic disorders characterized by marked skin and mucosal fragility, leading to blistering and erosions following minimal mechanical trauma. EB results from pathogenic variants in genes encoding structural components of the dermal–epidermal junction, including *KRT5* and *KRT14* (EB simplex), *LAMA3, LAMB3*, and *LAMC2* (junctional EB), *COL7A1* (dystrophic EB), and *FERMT1* (Kindler syndrome), among others. According to the level of tissue cleavage, EB is classified into four major types: EB simplex, junctional EB, dystrophic EB, and Kindler syndrome, each associated with distinct molecular mechanisms and clinical phenotypes.^(1-3)^

EB is a multisystem disorder with significant extracutaneous involvement, affecting the gastrointestinal tract, oral cavity, eyes, musculoskeletal system, kidneys, and cardiovascular system. Severe forms, particularly recessive dystrophic EB (RDEB) and junctional EB, are associated with chronic non-healing wounds, recurrent infections, malnutrition, progressive fibrosis, and a markedly increased risk of aggressive cutaneous squamous cell carcinoma, a leading cause of premature mortality in this population.^(4,5)^

Although the majority of EB research, clinical trials, and advanced therapeutic development remains concentrated in North America and Europe, Latin America has made relevant progress in genetic mapping, molecular diagnosis, and clinical characterization of EB patients. Regional reference centers and collaborative initiatives have contributed to improved genotype–phenotype correlations and the establishment of patient cohorts. Despite these advances, patients from Latin America still face substantial barriers to inclusion in global clinical trials and early access to innovative therapies. Ongoing regional efforts seek to integrate these patients into international research networks and to facilitate the introduction of new therapeutic solutions.

Over the past two decades, EB research has expanded considerably, encompassing basic disease mechanisms, wound healing biology, fibrosis, carcinogenesis, and pain and itch pathways. Translational efforts include gene replacement and gene editing strategies, RNA-based therapies, protein- and cell-based approaches, and the development of viral and non-viral delivery platforms.^(6-12)^ These advances have positioned EB at the forefront of cutaneous gene therapy research.

Clinical development has recently led to the approval of the first disease-modifying gene-based therapies for EB. However, these achievements have also highlighted profound global inequities. Vyjuvek™ (beremagene geperpavec), a topical gene therapy for dystrophic EB, was approved by the U.S. Food and Drug Administration in 2023, yet it remains unavailable to most patients in the Southern Hemisphere. Zevaskyn™ (prademagene zamikeracel), an autologous ex vivo cell-based gene therapy, is currently approved only in the United States and has an estimated cost exceeding three million US dollars per patient. In addition to cost, this therapy requires highly specialized manufacturing infrastructure, complex logistics, and prolonged hospitalization, representing major barriers to implementation in low- and middle-income countries. To date, scalable solutions enabling broad deployment of these therapies outside high-income regions remain limited.^(13-15)^

Promoting and valuing research in regions such as Latin America – through targeted funding, genuine international collaboration, capacity building, and regulatory alignment – represents a critical strategy to reduce these disparities. Facilitating access to therapies already approved in the United States and Europe may help bridge the gap between scientific innovation and patient benefit while strengthening local healthcare systems and regulatory pathways.^(16)^

Within this context, the EB2026 Congress was conceived as a platform to foster discussion on accelerating research, innovation, and access in EB. Its objectives included bringing together experienced investigators and researchers from other scientific disciplines, promoting interdisciplinary dialogue, and organizing forums involving clinicians, researchers, allied health professionals, and patient advocates. A central goal was to position the EB community as active protagonists in defining unmet needs and priorities, thereby contributing to the development of more inclusive, equitable, and globally relevant strategies for EB and other rare diseases.

## Data Availability

The content is already available.
